# Some New Lanoline Ointments

**Published:** 1889-08

**Authors:** E. Stern, Ernest Wende

**Affiliations:** Mannheim, Germany


### *SOME NEW LANOLINE OINTMENTS.*

By E. STERN, M. D., Mannheim, Germany. Translated from the *Therapeutische Monatshefte* for the Buffalo Medical and Surgical Journal by DR. ERNEST WENDE.

For the past three years, in continuation of experiments previously made, I have employed lanoline in the treatment of the various diseases of the skin, instead of the fats ordinarily used in compounding ointments. The following preparations will be characterized more especially for their newly-tested combinations:

### I.—SAPO-LANOLINE.

The so-called preparation consists of lanoline anhydricum and sapo-kalinus, first suggested by Prof. Liebreich as a substitute for mollin. The object is not to obtain the result of a soap containing an excess of fat, but simply an effect upon the integument from its recognized two-fold action. The mitigation of the soap with lanoline will be found an acceptable adjunct. The proportions which I generally order are sapo. two parts, lanoline two and one-half parts. Excepting salicylic acid, all usual medicaments such as boracic acid, tar, white precipitate, resorcin, etc., can be incorporated. The employment of ointments prepared in this way have the greatest advantage in the treatment of inveterate infiltrated eczemas, against all forms of dermatomycosis, as well as in cases of seborrhea, accompanied by an excessive formation of crusts and epidermis. In psoriasis capitis I most frequently apply the following formula:

| | |
|---|---|
| ℞—Hydrarg. precip. alba | 10.00 |
| Sapo. kalin. | 40.00 |
| Lanoline, anhydr. | 50.00 |
| M. | |

It is an established fact, in spite of the progress made in the therapy of psoriasis corporis since the introduction of the goa-preparations, the treatment of the psoriatic scalp has remained unchanged. Chrysarobin, apart from its usual ill-effects, does not in any way seem to influence the diseased scalp as favorably as the acknowledged process on the body. In two well-defined cases I failed to master this disorder, although the scalp, under proper precautions, was well rubbed in and thoroughly anointed three times weekly with a 25 per cent. ointment. The eruption on the body disappeared quickly upon a 20 per cent. application. Anthrarobin, recently introduced by Behrend, was found still milder in action, besides possessing the property of discoloring the hair. The result upon the head, with pyrogallic acid, is just as feeble as upon the skin elsewhere. Sapolanoline, impregnated with white precipitate, constitutes a remedy the effect of which upon the scalp is not less rapid or brilliant than that produced by chrysarobin upon the body. The accumulated crusts will disappear, after its application, from three to eight days. The patches will assume a smooth, white aspect. All that now remains necessary is the daily washing of the scalp, and the employment of an indifferent ointment, preferably lanoline cream, in order to relieve the slight tension and to bring the scalp back to its normal condition. I have never observed a discoloration of the hair with this method. The frequent reddening of the hair on using spirit saponat. is mostly due to the spirit.

### II.—LANOLINE UNGT. ADHESIV.

From the frequent occurrence of eczema of the head and face in children, the need of a means by which remedies might be held upon the skin without dressing, suggested itself to my mind. Owing to the cutting of pieces, their adaptation, and the application of a bandage, an advocate of the ointment dressings formerly in vogue will find that that method is exceedingly inexpedient and a waste of time. Neither do the pastes recommended during the last decade suit me.

They contain large quantities of powder. Although indifferent, they settle on a skin robbed of its epidermis. They are removed with difficulty, producing a mechanical irritation which does not compensate for the advantage of desiccation. It was only in chronic indolent catarrhal affections of the skin that I could testify with a certainty to a favorable result. In the construction of a fatty paste which is to adhere without further dressing, I have taken advantage of Lassar's idea, so beautifully demonstrated in his powder paste. Such a paste requires a temperature higher than the skin, and must be composed of perfectly indifferent fats or fat-like substances. This can be easily accomplished with a mixture of cera flava, lanoline, and some oil, as—

| | |
|---|---|
| ℞—Cer. flav. | |
| Lanolin, anhydr. | aa 40.00 |
| Ol. olivæ | 20.00 |

M. f. pasta usque ad refrigeret, agitand.

This preparation has a bright yellow color, and a thick, greasy consistence, resembling somewhat the sticking-wax of hair-dressers. It can be readily spread on the skin in rather thick layers, which adheres like a plaster, hence the term ungt. adhesiv. A hand rubbed with it appears as if it were covered with a kid glove.

This paste, which is perfectly neutral, is intended to serve as a basis. Most ingredients can be mixed with it without apparently altering its consistence. When tar is to be incorporated, the constituent of wax should be increased. Where an ointment dressing cannot be conveniently applied, I invariably use this paste either alone or in combination with boracic acid or zinc oxide,—especially in eczema of the face in children. Although somewhat slower in action, it renders the same service of protection, maceration and aid for the formation of epidermis. Generally after eight or fourteen days, depending somewhat upon the severity of the case, the application of tar may be commenced. I do not deem it necessary to speak of the advantage gained for both physician and patient with an application as convenient as this.

The addition of salicylic acid has a splendid effect in the treatment of squamous and vesicular varieties of eczema.

| | |
|---|---|
| ℞—Acid salicyl. | 3.00 |
| Ol. olivæ | 17.00 |
| Cer. flav. | |
| Lanolin anhydr. aa | 40.00 |

M. f. paste.

Salicylic acid, in this form without a cover, more or less imperme-able, displays its kereatolytic qualities in the mildest manner.

### III.—FLUID LANOLINE-INJECTIONS.

Upon the supposition that lanoline will adhere to mucous membranes, I concluded that by a liberal addition of oil it might be used as an injection. Owing to the property of absorbing water which cholesterin fat possesses, it was possible at the same time to inject remedies in solution. Salicylic acid was naturally dissolved in oil. Thus we have the formulas:

| | | |
|---|---|---|
| 1. | ℞—Lanolin anhydr. | 25.00 |
| | Ol. amygdal. | 75.00 |
| M. | (Basis-injection.) | |
| 2. | ℞—Zinc sulph. | 0.5 |
| | Aquæ | 4.5 |
| | Lanolin anhydr. | 20.0 |
| | Ol. amygdal | 75.0 |
| M. | | |
| 3. | ℞—Acidi salicylici | 0.25 |
| | Ol. amygdal | 75.00 |
| | Lanolin anhydr. | 24.75 |
| M. | | |

The injection is to be made with the ordinary p. p syringe.

The injection employed as a basis on being retained in the urethra for five to ten minutes has an exceedingly soothing effect, and lessens the irritation. Twenty-four hours later, fat globules may still be found in the urine. This long retention explains its favorable influence upon the gonorrheal process. I use the "basis-injection" in the first stage of gonorrhea. After eight to ten days, an antiseptic or an astringent drug is mixed with it, and I finally wind up with 1½ per cent. solution of resorcin in water.

Lanoline injections may also be employed to good advantage in chronic urethritis anterior.

This method has nothing in common with Tommasoli, who injects a solid ointment by means of a specially devised syringe.

---

A "PHYSICIAN of eminence," in London, is said to have called kissing one of the most common causes of the spread of disease, and to have also intimated that the practice itself is a disease. Indiscriminate kissing of babies and general promiscuous kissing is certainly dangerous and to be discountenanced, but we should dislike to see the "disease" altogether eradicated. Our theory is that we have to suffer a certain amount of disease in any event, and this being so, we prefer to acquire it in this way.—*Times and Register.*

